# Effects of ethyl pyruvate on cardiac function recovery and apoptosis reduction after global cold ischemia and reperfusion

**DOI:** 10.3892/etm.2014.1581

**Published:** 2014-02-24

**Authors:** JIALONG GUO, JUN ZHANG, XIANGYU LUO, WEIMIN LUO, CHENGYI LIN, KAILUN ZHANG, YANMEI JI

**Affiliations:** 1 Department of Cardiothoracic Surgery, Taihe Hospital Affiliated to Hubei Medical College, Shiyan, Hubei 442000, P.R. China; 2 Department of Cardiovascular Surgery, Union Hospital, Huazhong University of Science and Technology, Wuhan, Hubei 430022, P.R. China

**Keywords:** ethyl pyruvate, apoptosis, heart, rat, transplantation

## Abstract

The present study used an *in vitro* model of cold cardioplegia in isolated working rat hearts to evaluate the possible role of ethyl pyruvate (EP) in promoting cardiac function and preventing apoptosis. Two groups of rats were evaluated; the EP (2 mM EP; n=8) and control (n=8) groups. Isolated rat hearts were perfused with Krebs-Henseleit buffer (KHB) for 30 min, arrested with cardioplegic solution and stored for 4 h in B21 solution at 4°C. The hearts were reperfused with KHB for 45 min. EP was added to the cardioplegic and storage solutions and also to KHB for reperfusion. Cardiac parameters of the heart rate, including left ventricular systolic pressure, left ventricular end-diastolic pressure, left ventricular developed pressure and maximal rise rate of the left ventricular pressure, were monitored. In addition, coronary flow, adenosine triphosphate (ATP) levels and malondialdehyde (MDA) content were recorded and apoptotic cell determination was detected. The functional parameters in the EP group were significantly higher compared with those in the control group during the reperfusion period (P<0.05). In addition, ATP levels were higher in the EP group than in the control group and the content of MDA was lower in the EP group than in the control group. A concentration of 2 mM EP significantly reduced the number of apoptotic cells in the EP group compared with that of the control group (P<0.05). Therefore, EP significantly preserved cardiac function, enhanced tissue ATP levels, attenuated myocardial oxidative injury and markedly reduced apoptosis following myocardial ischemia in an *in vitro* model of 4 h of cold cardioplegia and reperfusion.

## Introduction

To improve the rate of successful heart transplantations, organ preservation should be optimized in cardiac transplantation surgery. However, the functional depression of cardiac grafts in postoperative recovery is not exceptional and the vitality of the transplanted tissue depends considerably on cardioplegic and storage conditions. At present, heart preservation is limited to 4–6 h of cold ischemic storage (1). Reperfusion injury occurs when there has been inadequate myocardial protection during the preceding ischemic period. Cardiac fatty acid and glucose metabolism are highly regulated processes that meet the majority of myocardial energetic requirements. Cardiac ischemia reperfusion (I/R) is characterized by complex alterations in fatty acid and glucose oxidation that ultimately have a negative impact on cardiac efficiency and function. Therefore, targeting metabolic events may be a promising strategy to reduce I/R injury (2).

Ethyl pyruvate (EP) is a key intermediate in the metabolism of glucose and is a potent reactive oxygen species (ROS) scavenger, which may promote the release of high-mobility group protein B1 (HMGB1). EP has been reported to inhibit myocardial apoptosis and reduce myocardial I/R injury in a variety of *in vitro* and *in vivo* model systems, including our previous study (3–5). During cardiac surgery and heart transplantation, cardioplegic arrest is used to protect the myocardium against the consequences of ischemia (6). When the heart is protected against ischemic injury by cardioplegic arrest, it is important to elucidate which additives have cardioprotective effects against I/R injury in the cardioplegic solutions. However, there are no data available on the effects of EP on cardiac function and apoptosis following prolonged cold ischemic conditions, including those used for heart transplantation. Therefore, it was hypothesized that EP may provide protection against reperfusion injury following prolonged hypothermic storage.

In the present study, isolated rat hearts were prepared similarly to those used for heart transplantation and were treated with EP before and/or after 4 h of global cold (4°C) ischemia. Hemodynamic parameters, adenosine triphosphate (ATP) levels, malondialdehyde (MDA) content and apoptotic cell determination were studied as the experimental variables. The aim of the present study was to determine whether the addition of EP to storage solutions and perfusion reduced the extent of reperfusion injury in the isolated rat heart.

## Materials and methods

### Animals

Adult male Wistar rats (weight, 220±30 g) were provided by the Experimental Animal Center of Tongji Medical College (Wuhan, China). All animals were treated in accordance with the Guide for Care and Use of Laboratory Animals published by the US National Institutes of Health. The study was approved by the ethics committee of Hubei Medical College (Shiyan, China). EP was purchased from Sigma-Aldrich Chemie (St Louis, MO, USA).

### Model of isolated and perfused working rat heart

Rats were anesthetized by intraperitoneal administration of 1 ml/100 g thiopental sodium and intravenous injection of 500 IU heparin. The chest was opened by bilateral sternocostal triangle and the hearts were immediately excised and placed into a cold bath (4°C) containing Krebs-Henseleit buffer (KHB; 11 mM glucose, 118 mM NaCl, 1.2 mM MgSO_4_, 25 mM NaHCO_3_, 1.2 mM KH_2_PO_4_ and 3 mM CaCl_2_). Hearts were fixed through the aortic root and left atrium on the perfusion cannulas of the Langendorff apparatus and perfused in Langendorff mode for 15 min (stabilization period) at a constant pressure of 70 cm H_2_O. KHB was used as a perfusion medium and saturated with 95% O_2_ and 5% CO_2_ (pH 7.4) at a stable temperature of 37°C. Hearts with a heart rate of <270 bpm were excluded from the study. At the end of the stabilization period, the perfusion mode was switched to the working heart mode for 15 min (WH-mode). The pressure in the left atrium was maintained at 10 cm H_2_O and fluid was ejected through the aortic root against a stable pressure of 80 cm H_2_O in the aortic cannula. After 15 min of perfusion in the WH-mode, the heart was arrested using 20 ml cardioplegic solution (St. Thomas’ solution; modified at 4°C; 114 mM Na^+^, 2 mM Ca^2+^, 20 mM K^+^, 203 mM Cl^−^ and 16 mM Mg^2+^) injected via the aortic cannula deviation under a pressure of 60 cm H_2_O. Hearts were disconnected from the circuit, immersed in storage solution (solution B21; 132.2 Na^+^, 0.9 Ca^2+^, 4 K^+^, 108.4 Cl^−^4 and 27.6 mM lactate) and stored in a cold box (4°C) for 4 h. After 4 h of cold ischemia, reperfusion at 37°C in the Langendorff mode for 15 min was established to stabilize the basic recovery conditions before the mode was switched to the WH-mode for 30 min. Throughout each experiment cardiac parameters, including the heart rate (HR), left ventricular systolic pressure (LVSP), left ventricular end-diastolic pressure (LVEDP), left ventricular developed pressure (LVDP = LVSP-LVEDP) and maximal rise rate of left ventricular pressure (+dp/dt_max_, −dp/dt_max_), were continuously monitored and recorded using a data acquisition system (PowerLab/8S; ADInstruments, Bella Vista, Australia). Coronary flow (CF) was measured by timed collection of the coronary effluent draining from the pulmonary artery cannula and was used as an index of vascular diastolic function. At the end of reperfusion, the left ventricle was quickly removed and stored in liquid nitrogen for additional assays.

### Protocols of perfusion

Two experimental groups were evaluated; the control (n=8) and EP groups (n=8). The hearts of the EP group received 2 mM EP, as described previously (7). The two groups underwent the same protocol with the exception that EP was added to the cardioplegic and storage solutions during ischemia and added to the KHB solution during reperfusion in the EP group.

### Measurement of myocardial ATP levels

ATP levels were quantified using the commercially available ENLITEN^®^ ATP Assay System (Promega Corp., Madison, WI, USA). At the end of reperfusion, the myocardial tissue specimens were immediately frozen in liquid nitrogen and individually pulverized into a fine powder by hand grinding with a dry ice-chilled steel mortar and pestle (8). Myocardium samples (10 mg) were homogenized with 1 ml precooled extractant (0.1% trichloroacetic acid) and centrifuged at 680 × g for 10 min (9). Supernatant (100 μl) was diluted 10-fold with 50 mmol/l Tris-acetate buffer containing 2 mmol/l EDTA (pH 7.75). Next, 100 μl sample extract or reference standard solution was placed in a tube luminometer (Turner Designs Luminometer TD-20/20; Promega Corp.), which was followed by the auto-injection of 100 μl ATP luciferin/luciferase assay mix for ATP quantification. Luminescence was measured at a set lag time of 1 sec and integration time of 10 sec.

### Measurement of myocardial MDA levels

The MDA assay method, as described by Yagi in 1976 (10), was designed to estimate the extent of oxidative damage. Heart bioptic samples (500 μl) were homogenized with 1 ml phosphate-buffered saline (PBS; 15 mM Na^+^ and 145 mM K^+^; pH 7) at 4°C and incubated with 1.5 ml thiobarbituric acid-reactive substances (TBARS). TBARS contained thiobarbituric acid (13.5 g; Sigma, St Louis, MO, USA), trichloracetic acid (TCA; 0.33 g) and hydrochloric acid (HCl; 8.5 ml) in 100 ml distilled water. Successive procedures included: i) Heating at 100°C for 15 min; ii) cooling and the addition of 1 ml TCA (70%); and iii) incubation for 20 min. Centrifugation was performed at 300 × g for 10 min. The MDA concentration was determined using a spectrophotometer (PerkinElmer LS-5; PerkinElmer, Inc., Norwalk, CT, USA) at 515 nm excitation and 535 nm emission. Results were expressed as μmol/g protein.

### Determination of myocardial apoptotic cells

Frozen sections from the left ventricle (5-μm thick) were fixed with 4% paraformaldehyde solution. To detect the extent of DNA degradation, the terminal deoxynucleotidyl transferase (TdT)-mediated biotin-dUTP nick-end labeling method was performed (*In situ* Cell Death Detection Kit, POD; Boehringer Ingelheim GmbH, Mannheim, Germany). Slides were incubated with proteinase (20 μg/ml in 10 mM Tris-HCl) for 20 min at room temperature (pH 7.4–8.0). Next, the slides were rinsed with PBS-blocking solution and incubated with permeabilization solution (0.1% Triton X-100 in 0.1% sodium citrate) for 2 min at 4°C. Following several washes with PBS, the samples were incubated with TdT and detection buffer conjugated with horse-radish peroxidase (Converter-POD) in a humidified chamber at 37°C for 60 min. For visualization, a diamino-benzidin-chromogen (Boehringer Ingelheim GmbH) was used and counterstaining with hematoxylin and eosin was performed. All experiments were performed according to the manufacturer’s instructions. To analyze the apoptotic cells, a light microscope was used (magnification, ×200). The apotosis index (AI) was calculated using the following formula: (Number of apoptotic cells/total number of cells counted) ×100. Quantitative analysis was performed by counting the cells in a randomly selected area of each tissue sample.

### Statistical analysis

All data are expressed as the mean ± SD. Analysis of variance with Tukey’s test was used to perform statistical analysis. P<0.05 was considered to indicate a statistically significant difference.

## Results

### Cardiac function parameters

**Figure 1:**
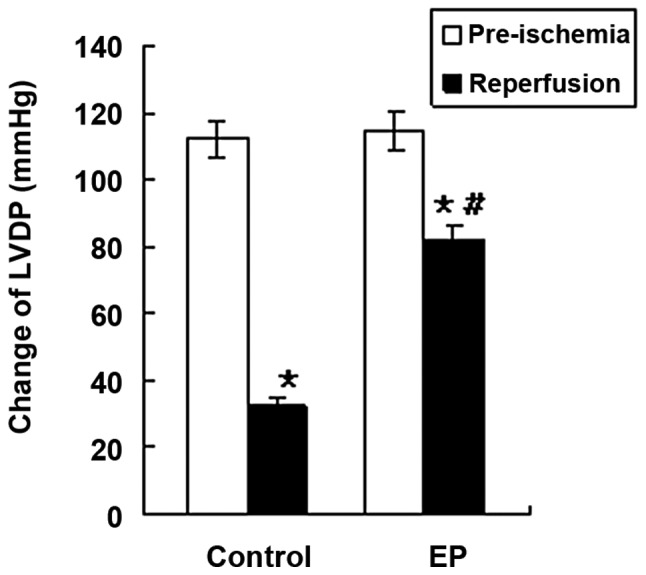
Effect of pre- and post-ischemic treatment with EP on the rate of recovery of LVDP during the reperfusion period after 4 h of global cold ischemia in isolated rat hearts. EP treatment during the reperfusion period significantly improved LVDP. Values are expressed as the mean ± standard error of the mean (n=8 per group). ^*^P<0.05, vs. respective pre-ischemia value; ^#^P<0.05, vs. control group. LVDP, left ventricular developed pressure; EP, ethyl pyruvate.

**Figure 2:**
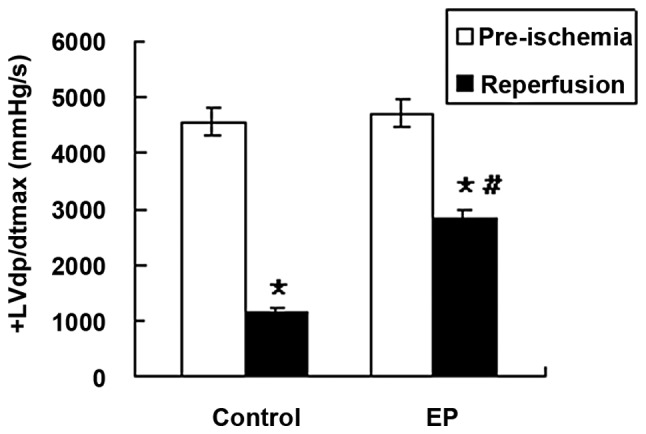
Effects of pre- and post-ischemic treatment with EP on the rate of recovery of +LVdp/dt_max_ during the reperfusion period after 4 h of global cold ischemia in isolated rat hearts. EP treatment during the reperfusion period significantly improved +LVdp/dt_max_. Values are expressed as the mean ± standard error of the mean (n=8 per group). ^*^P<0.05, vs. respective pre-ischemia value; ^#^P<0.05, vs. control group. +LVdp/dt_max_, maximal differentials of LVDP; LVDP, left ventricular developed pressure; EP, ethyl pyruvate.

There were no significant differences in the functional parameters between the control and EP groups in the period of pre-ischemia (Table I). The functional parameters, including LVDP, +LVdp/dt_max_, −LVdp/dt_max_ and CF, decreased significantly in the control and EP groups during reperfusion (P<0.05; Table I, Figs. 1 and 2), indicating the damaging effect of I/R on left ventricular function. The rats in the EP group exhibited a better recovery during reperfusion following ischemia than that of the control group. The functional parameters in the EP group were significantly higher compared with those in the control group during the reperfusion time (P<0.05; Table I, Figs. 1 and 2). No significant differences in HR were observed in all the rat hearts (Table I). Thus, the results indicated that EP increased the tolerance of the hearts to I/R injury.

### Myocardial ATP and MDA levels

**Figure 3:**
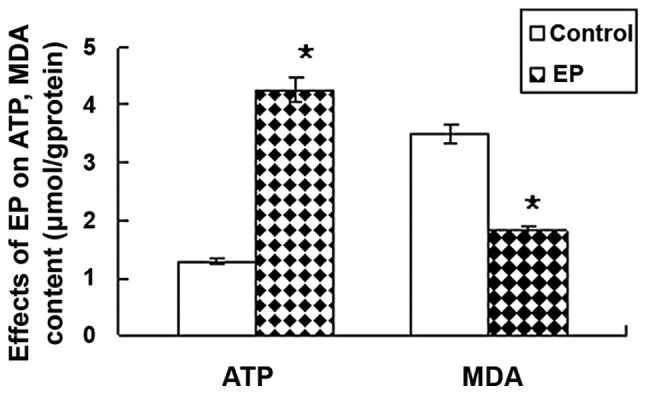
Effects of pre- and post-ischemic treatment of EP on ATP levels and MDA content during the reperfusion period after 4 h of global cold ischemia in isolated rat hearts. The levels of ATP were higher in the EP group than in the control group. The content of MDA was lower in the EP group compared with the control group. Values are expressed as the mean ± standard error of the mean (n=8 per group). ^*^P<0.05, vs. corresponding value of the control group. EP, ethyl pyruvate; MDA, malondialdehyde; ATP, adenosine triphosphate.

As shown in Table II, the levels of ATP (4.26±0.43 μmol/g protein) were significantly higher in the EP group than in the control group (1.28±0.17 μmol/g protein). The content of MDA was lower in the EP group (1.8±0.3 μmol/g protein) compared with the control group (3.5±0.5 μmol/g protein; P<0.05; Fig. 3).

### Apoptotic myocardial cells

**Figure 4:**
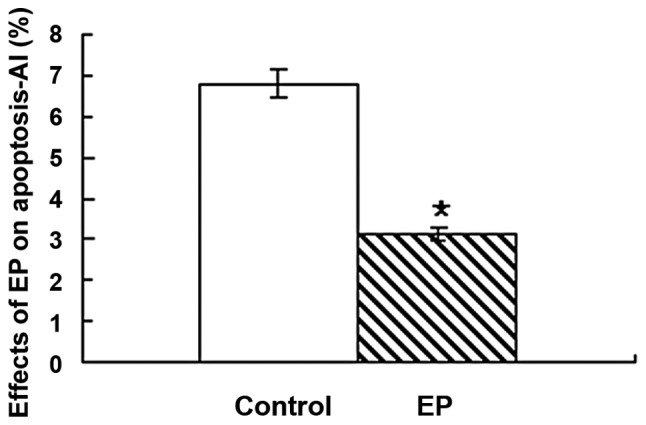
Percentage of nuclei staining positive for the TUNEL assay in heart tissue samples after 4 h of global cold ischemia and 45 min of reperfusion in the EP (3.1±1.2%) and control (6.8±1.6%) groups. EP, ethyl pyruvate; TUNEL, terminal deoxynucleotidyl transferase-mediated biotin-dUTP nick-end labeling.

Administration of 2 mM EP significantly reduced the number of apoptotic cells in the EP group (3.1±1.2%) when compared with the control group (6.8±1.6%; P<0.05; Table I and Fig. 4).

## Discussion

I/R injury of the myocardium is a significant entity in heart transplantation. Although numerous attempts to study the molecular interactions and elucidate the onset and time course of the functional alterations concerning I/R have been made in the previous two decades, the mechanisms of I/R remain unclear. Myocardial dysfunction and cellular injury occurs due to metabolic depletion during ischemia followed by ROS formation during reperfusion. Significant research efforts have investigated techniques of protecting the myocardium against I/R injury. The present study utilized EP as a myocardial protection agent and administered EP to isolated rat hearts and evaluated a possible role of EP in promoting cardiac function and preventing apoptosis. To the best of our knowledge, this study was the first to analyze the effects of EP in a cardiovascular model of 4 h of cold cardioplegia and reperfusion to mimic heart preservation in clinical heart transplantations.

EP parent compound, the glycolytic product pyruvate, is a natural metabolic fuel and antioxidant in the myocardium and other tissues, that exerts a variety of cardioprotective actions when provided at supraphysiological concentrations. Pyruvate increases the cardiac contractile performance and myocardial energy state, bolsters endogenous antioxidant systems and has been shown to attenuate myocardial ischemic injury through metabolic augmentation and antioxidant mechanisms. However, pyruvate is limited as a potential therapeutic agent due to extreme aqueous instability (11–13). EP is an ester derivative of pyruvate that is used as a food preservative and is highly stable in calcium containing solutions (14). A previous study demonstrated the ability of EP to enhance ATP levels, attenuate oxidative stress and preserve myocardial function in a model of prolonged myocardial I/R injury (15). EP has subsequently been studied in trauma, organ protection, critical care literature with models of organ ischemia, hemorrhagic shock and endotoxemic sepsis, all demonstrating a cytoprotective effect (16–20). An additional postulated role of EP is associated with its anti-inflammatory properties. *In vitro*, EP appears to directly inhibit nuclear factor-κB (NF-κB) and p38 mitogen-activated protein kinase pathways of inflammatory cytokine activation (21). Recently, Jang *et al* (3) reported that EP has the ability to inhibit neutrophil activation, inflammatory cytokine release and NF-κB translocation, which is associated with delayed myocardial protective effects following regional I/R injury in an *in vivo* rat heart model. In addition, Hu *et al* (5) reported that EP reduced myocardial I/R injury by inhibiting HMGB1 in rats.

In the present study, the addition of EP significantly prevented post-I/R injury and promoted cardiac function recovery in isolated rat hearts following 4 h of global cold I/R. A previous study demonstrated that EP scavenges the hydroxyl radical (^•^OH) and the effects are dose-dependent (22). These results may explain the better recovery of cardiac function with administration of 2 mM EP to the perfusion and storage solutions following cold global ischemia, since ^•^OH is considered to be the most cytotoxic oxygen free radical. ROS at reflow following ischemia may increase peroxidation of mitochondrial membranes and metabolic enzyme activities to prevent the recovery of heart metabolism and functional parameters. The decrease of free oxygen radical toxicity by free radical scavengers at the time of reflow improves the recovery of high-energy phosphate contents, indicating an association between oxygen free radical production and the impairment of myocardial energy metabolism during reperfusion (23).

The second mechanism of the protective action of EP in the heart may involve its own metabolism (24). EP is an important component of the energy chain in mitochondria and may restore oxidative metabolism. Furthermore, EP has a low molecular weight, which provides enough mobility to penetrate into cellular compartments, including the mitochondrial cytosol. It is possible that the protective effects are associated with other oxidizable energy substrates such as glucose (25).

To confirm the proposed mechanism of action of EP attributed to glycolytic substrate augmentation, tissue ATP levels were assayed. Excess exogenous pyruvate may liberate nicotinamide adenine dinucleotide and increase the proximal glycolytic pathway generation of ATP. Myocardial oxidative injury was diminished with EP. Compared with other inferential assays of free radical injury, including measuring MDA levels, the lipid peroxidation assay is a direct measure of free radical tissue injury. The reduction in lipid peroxidation in the EP group, when compared with that the control group, is an indication of reduced free radical injury.

A possible involvement of ROS in pathways promoting apoptosis is now widely accepted (26). Mitochondria and redox-state changes appear to have a predominant role in the promotion and expression of apoptosis (27). The intracellular changes during ischemia and reperfusion, including the accumulation of H^+^ and Ca^2+^, as well as the disruption of the mitochondrial membrane potential, result in the formation of free radicals or ROS. ROS accumulation and the subsequent activation of proinflammatory pathways are important in I/R injury (28). The resulting disturbances of metabolic processes can endanger cell existence due to the promotion of programmed cell death. The present study confirmed the presence of an increased number of apoptotic cells in a cold I/R injury model of isolated rat hearts. EP appeared to effectively reduce the extent of apoptosis similarly to other cardioprotective agents, including deferoxamine (29) and carvedilol (30). However, the precise mechanism that accounts for the reduction of apoptosis by EP requires further study.

A concentration of 2 mM EP was selected in the current study as previous studies with a Langendorff model had demonstrated that this concentration did not affect the basic cardiac function, but was capable of inhibiting the apoptosis of cardiac myocytes (4). The timing of EP administration was designed to enhance the two purported mechanisms of action, glycolytic substrate augmentation and antioxidation. However, further studies are warranted. Future investigations should evaluate the myocardial protective capacity of EP in other models of myocardial ischemia as a means of broadening the spectrum of clinical utility. To closely mimic the typical ischemia that occurs during heart transplantation, an *in vivo* animal model should be engaged to appraise the protective effects of EP in the future. In addition, future studies should determine whether using higher doses of EP yields even greater myocardial protective effects. At present, only a limited dose-response curve of three concentrations of EP spanning 3 logs has been studied (31). Alternate routes of administration, particularly intracoronary, should also be evaluated to search for increased efficacy.

In conclusion, EP significantly preserves cardiac function, enhances tissue ATP levels, attenuates myocardial oxidative injury and markedly reduces apoptosis following myocardial ischemia, as shown in a cardiovascular model of 4 h of cold cardioplegia and reperfusion.

## Figures and Tables

**Table I:** Hemodynamic variables.

Variables	Control group (n=8)	EP group (n=8)
Pre-ischemia		
LVDP (mmHg)	112.3±14.2	114.6±12.1
LVEDP (mmHg)	12.4±1.8	11.8±1.6
+LV dp/dt_max_ (mmHg/sec)	4 562±574	4 727±548
−LV dp/dt_max_ (mmHg/sec)	−2 548±316	−2 436±280
HR (beats/min)	223±26	228±24
CF (ml/min)	12.7±1.8	12.5±1.6
Reperfusion		
LVDP (mmHg)	32.7±5.1^a^	82.4±7.5^a^,^b^
LVEDP (mmHg)	65.7±8.3^a^	30.3±4.5^a^,^b^
+LV dp/dt_max_ (mmHg/sec)	1 175±153^a^	2845±367^a^,^b^
−LV dp/dt_max_ (mmHg/sec)	−786±104^a^	−1425±164^a^,^b^
HR (beats/min)	232±56	228±53
CF (ml/min)	3.8±0.5^a^	8.2±1.0^a^,^b^

Data are expressed as the mean ± SD.

a P<0.05, vs. respective pre-ischemia value;

b P<0.05, vs. control group.

LVDP, left ventricular developed pressure; LVEDP, left ventricular end-diastolic pressure; ±LV dp/dt_max_, maximal differentials of LVDP; HR, heart rate; CF, coronary flow; EP, ethyl pyruvate.

**Table II:** Measurement of ATP, MDA content and AI.

Group	ATP (μmol/g protein)	MDA (μmol/g protein)	AI (%)
Control	1.28±0.17	3.5±0.5	6.8±1.6
EP	4.26±0.43^a^	1.8±0.3^a^	3.1±1.2^a^

Data are expressed as the mean ± SD (n=8 per group).

a P<0.05, vs. control group.

ATP, adenosine triphosphate; MDA, malondialdehyde; AI, apoptosis index; EP, ethyl pyruvate.
